# Impact of a prehabilitation and recovery programme on emotional well-being in individuals undergoing cancer surgery: a multi-perspective qualitative study

**DOI:** 10.1186/s12885-023-11717-1

**Published:** 2023-12-14

**Authors:** Rachael Powell, Amy Davies, Kirsty Rowlinson-Groves, David P. French, John Moore, Zoe Merchant

**Affiliations:** 1 Manchester Centre for Health Psychology, Division of Psychology and Mental Health, School of Health Sciences, University of Manchester, Manchester, M13 9PL UK; 2 Division of Nursing, Midwifery & Social Work, University of Manchester, Manchester, UK; 3 GM Active, Salford Community Leisure, Manchester, UK; 4 Department of Anaesthesia, Manchester Royal Infirmary, Manchester University NHS Foundation Trust, Manchester, UK; 5 Greater Manchester Cancer Alliance, The Christie NHS Foundation Trust, Manchester, UK; 6 Division of Diabetes, Endocrinology and Gastroenterology, School of Medical Sciences, University of Manchester, Manchester, UK; 7 North West Lung Centre, Lung Cancer and Thoracic Surgery Directorate, Wythenshawe hospital, Manchester University NHS Foundation Trust, Manchester, UK

**Keywords:** Cancer, Prehabilitation, Rehabilitation, Anxiety, Self-efficacy, Surgery, Psychological support, Well-being, Psychological stress, Confidence

## Abstract

**Background:**

Prehabilitation and recovery programmes aim to optimise patients’ physical fitness and mental well-being before, during and after cancer treatment. This paper aimed to understand the impact of such a programme on emotional well-being in individuals undergoing cancer surgery. The programme was multi-modal, containing physical activity, well-being and nutritional support.

**Methods:**

Qualitative interviews were conducted with 16 individuals who participated in a prehabilitation and recovery programme. Twenty-four health care staff involved in referral completed an online survey. An inductive, thematic analysis was conducted, integrating perspectives of patients and staff, structured with the Framework approach.

**Results:**

Patients seemed to experience emotional benefits from the programme, appearing less anxious and more confident in their ability to cope with treatment. They seemed to value having something positive to focus on and control over an aspect of treatment. Ongoing, implicit psychological support provided by Exercise Specialists, who were perceived as expert, available and caring, seemed valued. Some patients appeared to appreciate opportunities to talk about cancer with peers and professionals. Discomfort with talking about cancer with other people, outside of the programme, was expressed.

**Conclusions:**

Participation in a prehabilitation and recovery programme appeared to yield valuable emotional well-being benefits, even without referral to specialist psychological support.

**Study registration:**

The study protocol was uploaded onto the Open Science Framework 24 September 2020 (https://osf.io/347qj/).

**Supplementary Information:**

The online version contains supplementary material available at 10.1186/s12885-023-11717-1.

## Background

About half of people with cancer experience distress which negatively impacts quality of life at diagnosis, with many continuing to experience anxiety and depression over subsequent months [1]. One multi-centre study identified ‘clinically significant’ distress in 52% of respondents with cancer [2]. For many individuals, psychological support could be beneficial, but emotional difficulties may be missed by health professionals. Further, there is a shortage of suitably skilled health professionals to provide specialist support [1].

Emotional well-being is particularly pertinent for individuals undergoing surgery as higher pre-operative anxiety has predicted worse post-operative functional limitations and pain in wider surgery contexts [3, 4]. A systematic review found that interventions incorporating psychological strategies to prepare people for surgery may reduce pain, length of stay and negative emotion post-surgery [5].

Increasingly, ‘prehabilitation’ interventions are being implemented with the primary aim of optimising physical function before cancer treatments such as surgery. Such interventions may also include ‘rehabilitation’ elements to aid recovery and optimise future health [6]. Typically, physical fitness training is a key element of such interventions, but they may also include nutrition and mental health support [6]. Notably, psychological support is recommended as a component of prehabilitation in recent guidance [7–9]. A ‘review of reviews’ has identified potential for psychological prehabilitation interventions to enhance well-being but identified methodological and reporting issues such that clear conclusions could not be reached regarding what exactly might be effective [10].

‘Prehabilitation’ interventions may also yield well-being benefit via several mechanisms without use of explicit psychological support components. Qualitative study reports have identified potential mechanisms including having something to focus on away from cancer concerns [11–13] and a sense of active involvement in, and control over, an element of their health care [13, 14]. Individuals may also benefit from receiving social support from peers at programme sessions [12, 14, 15]. More generally, physical activity interventions have been demonstrated to yield emotional benefits in various populations [16].

However, it is not clear exactly what individuals might find beneficial (or detrimental) for their emotional well-being, or why. The present paper reports findings from within a wider qualitative, multi-perspective study. The wider study aimed to understand how patients undergoing surgery for cancer perceived a prehabilitation and recovery programme, and focussed on issues affecting acceptability, engagement and referral. Those findings are reported elsewhere [17]. The current report presents findings related to the emotional well-being impact of a prehabilitation and recovery programme that were not fully anticipated in the original research plan.

## Methods

### Design

Single qualitative interviews were conducted by phone or video call with individuals who engaged with an exemplar prehabilitation and recovery programme. An online questionnaire was completed by healthcare staff involved in referral of patients to the programme - ‘clinician’ participants. The study used methods also reported in Powell et al. [17].

### Setting

Data were collected in the context of the Greater Manchester Cancer Alliance Prehab4Cancer and Recovery Programme (‘the programme’), an award-winning prehabilitation and recovery programme within a large conurbation with multiple National Health Service (NHS) hospitals in the North West of England. Key aspects of the programme are described in Table 1; further information about the programme, including details of exercise intensity and type, is provided in detail elsewhere [6]. Individuals with colorectal, lung or oesophago-gastric cancer were offered supported physical activity before, during and after treatment. Mental wellbeing was assessed; concerns were raised with clinical teams and patients were referred to mental health support services where appropriate. Nutritional status was also assessed and supported. Programme instructors were Exercise Specialists; they received training designed to enable staff with various roles to provide effective basic psychological support to patients [18]. This training is designed to meet ‘Level 1’ standards, providing staff with ‘general emotional care’ skills and competence to identify (and refer for) psychological needs requiring specialist input [1]. They also received regular reflective practice, continued professional development, oversight and advice from a cancer specialist Clinical Psychologist and a highly specialist Occupational Therapist, dual trained in mental and physical health qualified at Level 2.

**Table 1:** Summary of some key features of the Greater Manchester Cancer Alliance Prehab4Cancer and Recovery Programme

Aspect of programme	Details
Assessment	• Patients assessed at 4 time points (baseline, pre-operatively, post-operatively and end of rehabilitation). • Under COVID restrictions (from March 2020), assessments were conducted by phone and/or video call. • Patients were allocated to ‘universal’ (for more fit individuals) or ‘targeted’ (for less fit individuals) arm of the programme.
Exercise provision	• Tailored exercise programme provided cardiovascular and resistance components; all participants received free gym membership. • Individuals on ‘universal’ programme were encouraged to attend gym at least 3 times/week; monitored by Exercise Specialists and supported where needed. • Individuals on ‘targeted’ programme were encouraged to attend 3 supervised sessions/week. Supervision was provided by Prehab4Cancer programme Exercise Specialists or gym instructors who supervised Prehab4Cancer participants alongside other individuals with long-term health conditions (attending via GP referral schemes). • Exercise duration: 45–60 min depending on level of fitness. • Under COVID restrictions, timetables for home exercise were provided; aim: minimum 3 sessions/week, with support provided via telephone/video calls. Online classes were arranged providing a supervised aspect; follow-along youtube videos were also provided.
Group composition	• Group or individual gym sessions; group online classes; individual monitoring and support. • Group sessions included a mixture of individuals in pre- and post-operative phases.

GP = General Practitioner

### Participants

***Patient participants*** were individuals who received surgery for cancer May 2019 - March 2020, were referred to the programme, were over 18 years old, and could speak and understand English. We aimed to gather sufficient data to gain a range of perspectives whilst being able to conduct a careful, in-depth analysis of issues which appeared to be of importance to participants. Exclusion criteria were: found to be unsuitable for the programme when assessed; change in diagnosis; immediate surgery (no time to take part in the programme). The wider study within which the present analysis was conducted aimed to ensure that individuals living in lower socio-economic status areas were included within the patient sample [17]. The wider study also recruited individuals who did, and did not, take part in the programme (‘engagers’ and ‘non-engagers’), intending to recruit approximately 15 participants in each group [17]. The present report focusses on experiences of ‘engager’ participants because it explores the emotional well-being impact of taking part in the programme – an experience that only ‘engager’ participants had.

KRG, a member of the programme delivery team during the running of the study, organised identification of individuals who met study inclusion criteria and the postage of invitation packs between October and December 2020. Initially, packs were mailed to individuals living in neighbourhoods within the three most deprived deciles, determined using the English Indices of Deprivation online tool [19]. Individuals living in other neighbourhoods were then mailed invitation packs, with those who had surgery most recently contacted first. Individuals who were interested in taking part contacted a University-based researcher.

***‘Clinician’ participants*** were healthcare professionals or other NHS members of staff involved in referral of patients to the prehabilitation and recovery programme. In the wider study, perspectives from staff were primarily sought to understand barriers in referring patients and staff perceptions of the prehabilitation and recovery programme [17]. However, staff inclusion also provided an opportunity to gain their perceptions of patients’ experiences with the programme. Approximately 200 individuals had referral-related roles; if 30% were to respond there would be a sample of 60 staff members.

ZM, a member of the programme project team, emailed key clinical contacts who were asked to share emails with all staff with referral roles. An advert was also placed on an online forum and on Twitter. Study information and the survey were available via a weblink from study adverts.

### Data collection

***Patient participants*** individually took part in an interview conducted by phone or video call with a University-based researcher. Informed consent was taken verbally before commencing the interview, and audio-recorded as a different file to the interview. Interviews were guided by an interview schedule that was developed by the research team with input from public involvement contributors. Participants were asked about experiences with the programme, acceptability, and barriers to participating. Emotional well-being impact was not a primary focus of the interview schedule, but questions about well-being support were included (Table 2). The full interview schedule is available elsewhere [17]. Interviews were conducted October 2020-January 2021 and audio-recorded. Audio-recordings were transcribed and identifying details removed.

**Table 2:** Examples of questions asked in interviews with patient participants

Topic	Example questions
Deciding whether to take part	What did you hope to gain from taking part? Did you have any concerns about taking part?
Experiences of the programme	What did you think about the exercise programme? What did you like/dislike about it? How did you find the first assessment/exercise sessions? Were there any problems or difficulties in taking part in the programme? What would have made it easier for you to take part? What did you think of your trainer? Do you feel you benefited from the exercise programme at all? How effective did you feel the exercise programme was in preparing you for surgery?
Well-being support	Did you receive support about coping or stress? Or to help you if your mood has been affected? How did you find this support? (e.g. how did you feel about it? How useful did you find it?)

***‘Clinician’ participants*** completed an online survey which was hosted on SelectSurvey November 2020 - January 2021. Informed consent was given by agreement with a consent statement on the online survey cover page. The survey was developed by the research team, including team members with clinical backgrounds. To minimise burden, a combination of categorical response and free-response boxes were used. Survey topics included staff members’ perceptions of the programme and their perceptions of patient experiences within the programme; demographic and work-related details were also requested. Example questions are provided in Table 3; the full survey is available elsewhere [17]. Responses were downloaded, checked to ensure that identifying details were not included, and securely stored.

**Table 3:** Examples of questions in ‘clinician’ survey

Question	Response options
How valuable do you think taking part in Prehab4Cancer is for patients? Please elaborate on your answer if you are able to do so	Extremely valuable / very valuable / quite valuable / not very valuable / not at all valuable Free response box
What do you think the benefits of Prehab4Cancer are for patients? (please tick all that apply)	• Improved fitness • Quicker recovery post-surgery • Fewer complications post-surgery • Improved long-term physical activity levels • Improved long-term health or fitness • Meeting people • Other (please state)
Have you received any feedback about Prehab4Cancer from patients?	Yes / No
If yes, what have patients said?	Free response box

### Analysis

An inductive thematic analysis was conducted aiming to identify and understand patterns within the dataset [20, 21]. We aimed to understand perspectives of both patient and ‘clinician’ participants, and conducted a multi-perspective analysis, integrating data from the two participant groups. The analysis was structured using the Framework approach [21, 22]. The analysis was conducted by two university-based researchers (RP and AD), with all other members of the research team contributing to the analysis in discussions of a draft working analytic framework and preliminary findings. See Appendix (Supplementary Material) for analysis details, including reflection on the various roles of research team members within the analysis process. The analysis was initially intended to focus on issues related to programme acceptability, engagement and referral, and the themes related to this focus are presented elsewhere [17]. The present report focusses on the emotional well-being impact of participating in the programme. Despite this area having little focus in the interview schedule, it was prominent in participants’ accounts of their experiences.

## Results

### Description of participants

Study packs were sent to 105 ‘engager’ and 103 ‘non-engager’ patients. There were twenty-five responses: two indicated intended recipients were deceased; two declined participation; two were not eligible for inclusion; one cancelled an interview (health issues). Interviews were conducted with 18 patient participants; 16 of these engaged with the prehabilitation and recovery programme and were included in the present findings (Table 4). Twenty-four eligible responses to the online survey were received from ‘clinician’ participants (Table 5); one further individual completed the survey but did not meet inclusion criteria.

**Table 4:** Patient participant information

	Participants (n or information) Total n = 18*
Diagnosis	
Bowel/colon cancer	9
Lung cancer	7
Oesophago-gastric cancer	2
Age (years)	Median 68.5; range 40s to 80s
Gender	
Female	9
Male	9
Ethnicity	
White British	16
Other ethnic group	2
Socio-economic status	
IMD score 1–3	9
IMD score 4–6	5
IMD score 7–10	4
Employment	
Retired or unemployed	15
Employed	3
Interview duration (minutes)	Median 43; range 29–99
Interview medium	
Phone Videocall	15 3
Participation in P4C Programme	
Engager	16
Non-engager	2

*The present report focuses on the 16 ‘engagers’ data. Demographic information is presented for all 18 patient participants (including ‘non-engagers’) to minimise risk of participant identification in study reports

IMD = Index of Multiple Deprivation. IMD score 10 = least deprived locality; IMD score 1 = most deprived locality

P4C Programme = the Prehab4Cancer and Recovery Programme

**Table 5:** ‘Clinician’ participant information

Characteristics (n reporting information)†	Participants (n or information)
NHS role (22)	
Nurse	11
Doctor	7
Other	4
Role in referral pathway (22; some had > 1 role)	
Directly refer patients	16
Input into referral decision	10
Introduce patients to the programme	2
Time since qualification (years) (20)	Median 19.5; range < 5 to > 35
Time involved in P4C referral pathway (months) (22)	Median 18; range < 5 to > 35
Age (years) (19)	Median 44; range 30s to 50s
Gender (20)	
Female	16
Male	4
Ethnicity (21)	
White/White British	20
Other ethnic group	1

**†**Total n = 24; n reporting demographic details varied

P4C Programme = the Prehab4Cancer and Recovery Programme

Participant recruitment was affected by COVID-19; the recruitment period coincided with restricted working practice and limited capacity for health service staff. Recruitment of healthcare staff was particularly impacted as the researcher could not visit referring teams and some planned reminders were not sent to avoid adding to workload pressures. The ‘clinician’ sample was smaller than initially expected, but thoughtful responses were received, and the range of participant roles suggested we gained views from a broad cross-section of staff.

### Analytical findings

Three analytical themes were developed relating to the emotional well-being impact of the programme, using data from both patient and ‘clinician’ responses: Emotional benefits of participation; Feeling looked after and Talking about cancer.

#### Emotional benefits of participation

Emotional benefits of taking part in the programme were perceived by both patient and ‘clinician’ participants:

*And I think just my general kind of mood as well, no doubt about it, was much better. (Patient H)*

*Patients appear to be in a better physical and psychological state with a more positive outlook (Clinician 3)*.

There appeared to be various ways in which benefits were experienced. For some individuals, the programme seemed to yield emotional benefits by providing something to focus on alongside the cancer diagnosis, during a time when a cancer diagnosis and related health issues could be felt to dominate:

*the exercise took a lot of the fear away of the word [‘cancer’], and the experience of it. And because I was thinking about my exercise and my fitness, it tended to take away quite a lot of the anxiety that I had.* [and later:] *It’s just – it focuses your mind away from just the fact that you’ve got a cancer. (Patient M)*

Some seemed to value an opportunity to gain a sense of control over their cancer experience:

*it’s very easy when you get the diagnosis to feel like a rabbit caught in headlights. For obvious reasons, the process moves very fast, and you’re like, “oh good heavens”, you know. But that gives you a feeling of time to take control, and I’m a control freak so that’s an important aspect for me. I felt I was actually doing something to help myself get through the journey. (Patient F)*

Participating in prehabilitation seemed to affect patients’ perceptions that they would be able to cope, physically and mentally, with surgery and recovery, and to reduce their anxiety regarding surgery:

*it gives you more confidence in your ability, not to worry, it’s all going to be fine. […] It just gives you like a mental strength of what’s to come. And yeah I can do this, I can cope with this*. *(Patient J)*

*I felt […] the operation wasn’t going to take as much out of me because I’m a bit fitter. I felt I was going to be home quicker. So, you know, those were big psychological boosts for me. (Patient O)*

The programme provided opportunities for individuals to learn from the experiences of other patients. Knowing that others undergoing similar procedures had made good recoveries seemed to enhance confidence in ability to cope, and lessen worries:

*I met [another patient] […] talking to her made me feel better. […] I thought well she got through it alright, very much the same and she’d been doing the prehab stuff (Patient E)*.

#### Feeling looked after

Patient participants were consistently highly positive about programme Exercise Specialists, describing them using terms such as: *‘brilliant’; ‘lovely’; ‘excellent’*. The team appeared to be widely perceived as authentically friendly and caring, taking time to support and encourage individuals:

*[the Exercise Specialist] was absolutely superb. Very warm, very understanding and said an awful lot of things that helped me really and gave me confidence. (Patient C)*

Within interviews, patient participants were asked about their experiences of psychological support provided within the programme. Some indicated that they did not feel they required such support, although support being available if required seemed welcomed:

*thankfully I didn’t personally need it. But, you know, it’s there if it is needed and it were nice to know that it were there, you know. (Patient L)*

Nevertheless, it seemed that some individuals who might feel uncomfortable about seeking or receiving explicit psychological support valued the more implicit emotional support routinely provided by Exercise Specialists:

*people go, “do you want somebody to talk to”, and the answer is always no [laughs]. But you didn’t feel like you were bullied into that sort of psychological support, it was just there without being labelled […] have they provided psychological support? Absolutely, no doubt about it. Have they ever called it that? No. […] they’ll just kind of go “how are you doing, you’re doing too much or too little or whatever”, so yeah I think it’s been superb. And the thought of having to go and speak to somebody about how I’m feeling is just, it’s not going to happen so this [laughs] – this works, yeah, this works (Patient H)*.

For some participants, their Exercise Specialist seemed to provide support throughout the treatment pathway which health care teams were seen as being unable to manage. Exercise Specialists were perceived as having both expertise and availability to discuss issues:

*[the Exercise Specialist] had more time to talk to me – when you’re at the hospital, don’t get me wrong, they’re absolutely fantastic but you are aware that they’re absolutely chock-a-block. Whereas the trainer’s got a lot more time, and they’ve got their degrees, they know what they’re talking about […] And I think her down to earth approach and having a bit more time to discuss things made a big difference to my state of mind (Patient F)*.

Patient participants seemed to value Exercise Specialists being accessible and responsive:

*And if you can’t get in touch with one, you’ve always got another one to go to that you can find out more information from. […] if you’ve got any problems, they will help you out and answer any questions but if, you know, if they can’t, or they’ll find the answer for you (Patient B)*.

The contact between patients and Exercise Specialists seemed two-way, with patients feeling able to contact Exercise Specialists, and Exercise Specialists also regularly contacting patients, which seemed to contribute to their feeling looked after and supported:

*I texted him after I’d had surgery and he kept in touch with me which I found was very good, just to say, how are you doing? Up until I went back. (Patient C)*

Overall, the approach of Exercise Specialists on this programme appeared to be important to patients, and seemed to ensure that individuals felt supported, without needing to seek or receive explicit psychological support. ‘Clinician’ responses seemed in agreement with this perspective:

*Comments [from patients] are generally very positive - the trainers are approachable and the extra contact they provide is perceived as beneficial. (Clinician 1)*

#### Talking about cancer

An issue apparent in some interviews was that people can feel uncomfortable with talking about cancer. There were individuals who felt that others were reluctant to talk about cancer with them – a sense that cancer is a taboo topic:

*For example, in the pub, you don’t want to be boring everybody about your anxiety or anything like that, or your workmates. People don’t want to hear. I noticed, with several people, you know, when they found out when I had cancer – I don’t think it would have been worse if you’d had the clap [slang: gonorrhoea], they seem to try and avoid you. So, yeah, I think for somebody to be able to, if they had any anxieties, have someone who they can talk to, who can deal with it in a professional manner […] some people are brilliant listeners, but other people I think don’t know how to handle it if someone tells them that they’ve got a problem […] I think it frightens people. (Patient I)*

*Otherwise I wouldn’t have spoken to anyone about it, you know, it’s not something you’d talk about. And I think – I think above all, these are all people who’ve had similar sort of things or about to have similar sort of things and it’s hard to get through the stages, so, you know, you’re talking about things like that. […] it’s different to talking to people you know, it’s more awkward, I think [and later:] I don’t think until – in actual fact prehab for cancer was probably the first time I’d really heard the word cancer because you don’t talk about it like that. (Patient E)*

These individuals seemed to value having access to individuals with whom they felt able to talk about cancer, whether those others are professionally trained (Exercise Specialists) or peers on the programme.

In some instances, patient participants appeared uncomfortable about being open with others about having cancer:

*Well, I think with what you’re going through at the time, you don’t want everybody in the world to know about it. (Patient R)*

*I was very determined that I wouldn’t want to be known as that person who was diagnosed with cancer […] I didn’t want people feeling sorry for me (Patient H)*.

Both of these participants seemed to value maintaining privacy around their diagnosis. Patient H’s quote suggests a fear of being pitied may underlie this perspective for some individuals. Nevertheless, it seemed that taking part in the programme actually helped Patient H to manage others’ responses to their illness, and to avoid pity.

*[…] helped me face the world a bit more, and helped me to talk about it a bit more. […] and to stop people feeling sorry for me as well, because I’m like look at me I’m exercising all this time, […] they keep making me do all this stuff, and look at how much fitter I was. So it’s good [laughs], to a certain extent stopped people going “oh poor you”, you know, which helped. (Patient H)*

It seemed that associating ‘cancer’ with gyms and exercises, both of which have healthy connotations, may have taken some of the fear of ‘cancer’ away for other people, leading to conversations about cancer being easier.

## Discussion

Participants identified perceived emotional well-being benefits of taking part in a prehabilitation and recovery programme, including having a positive occupation to focus on, gaining a sense of control, increased confidence in ability to cope with treatment and reduced anxiety. The day-to-day, implicit psychological support provided by Exercise Specialists seemed highly valued and acceptable to individuals who did not wish to receive explicit, specialist psychological support. Having professional support throughout treatment and recovery seemed appreciated. The programme appeared to provide an environment where participants could feel comfortable to talk about cancer with peers and/or programme staff. This seemed important for some individuals who felt uncomfortable about talking about cancer with others, perceiving that others can experience discomfort around discussing cancer.

These findings regarding emotional well-being benefits are consistent with those identified in other studies. Having something other than cancer concerns to attend to, and perceiving benefits of being actively involved in, and gaining a sense of control over, the cancer process have been reported elsewhere [11–14, 23]. However, there were areas where the present paper extends previous findings.

Cancer treatments can be highly invasive, and undergoing those procedures can be daunting. The present findings suggest that involvement in a prehabilitation programme may help to reduce such anxieties and increase individuals’ confidence that they can cope with treatment. Individuals may feel themselves to be in a better physical condition to manage treatment, and may learn from the experiences of others who have undergone, and coped with, similar treatment. In the surgery context, reduced anxiety may have particular benefits as it has been associated with better post-operative outcomes [3, 4]. Increased confidence in coping may also have broader benefits: higher self-efficacy has predicted lower distress and higher quality of life in cancer patients [24].

Recent guidance recommends including psychological support within prehabilitation [7, 8]. The programme considered in the present study included a mechanism for providing explicit, specialist support where needed by participants. However, for some participants, it seemed that implicit, ongoing, general emotional support may be more acceptable than referral to specialist psychological support or therapy. Reasons for not seeking psychological support may include focussing on ongoing day-to-day living rather than the illness, thinking that they are not struggling enough to need help, and seeing help-seeking as indicative of failure to cope [25]. A recent Australian qualitative study identified that individuals who had completed cancer treatment seemed to value the aspect of having ‘somebody in their corner’ – in this case support from a health coach as part of a ‘cancer survivorship programme’ [26]. Support provided by the health coach included general emotional support for emotional well-being, anxiety and depression, with the option of referral to more specific mental health support where psychological distress was identified. This Australian study’s findings included reports of individuals seeking participation in the programme because they desired emotional well-being support but were concerned about stigma or costs associated with seeing psychologists [26].

Our findings suggested that individuals can feel a sense of taboo, of feeling unable to talk about cancer because of others feeling uncomfortable with such discussions. Other researchers have reported individuals expressing reluctance to talk about cancer with family members and friends out of a desire to avoid burdening them, and wanting to minimise worry in close others [25, 27]. Prehabilitation and recovery programmes may provide an environment where cancer is the common denominator, enabling individuals to talk about their cancer experiences.

### Strengths and limitations

Whilst experience of well-being support was briefly raised in the interview schedule, the emotional well-being impact of participating in a prehabilitation and recovery programme was not intended to be the main focus either of the interview or the analysis. The clear importance of psychological support and emotional well-being benefit to patient participants identified despite this emphasises the significance of the present findings.

A further strength of the present study is the effective use of purposive sampling ensuring inclusion of individuals from locations with varying social economic status. However, the sample lacked ethnic diversity. This is problematic as experiences of services related to mental health can vary with ethnicity [28]. It may be, therefore, that important issues were missed and future research should seek understanding of individuals’ experiences of prehabilitation and recovery programmes across ethnic groups. As is typical of qualitative research, the aim of the present study was to gain insight and understanding of individuals’ experiences rather than to generate findings which are representative of the wider population, and caution is needed when considering application of findings. Nevertheless, the present findings largely complement and extend – rather than contradict – previous findings, which would seem to support their validity.

### Implications

The support provided by a prehabilitation and recovery programme seemed to help patients to cope with their treatment, ensuring that they felt looked after and providing valued opportunities to talk about their cancer experience. Programmes may benefit from being designed with these features in mind.

The Exercise Specialists in this programme had only received basic, Level 1 training in psychological support focused on ‘general emotional care’ [1, 18]. NICE guidance suggests that Level 1 psychological support could reduce the risk of individuals developing more serious problems, and as a result could reduce demand for higher level support [1]. The present study was not designed to test this hypothesis, but the findings were consistent with the concept that access to Level 1 support may yield benefits. Nevertheless, we would not advocate reduction in availability of specialist psychological support: the present study’s participants seemed pleased that such support would be available should they need it. Further, appropriate training and support of staff delivering programmes appears to be important: health coaches delivering an Australian cancer survivorship programme seemed concerned that they lacked relevant skills in providing emotional well-being support to individuals who have received cancer treatment [26].

## Conclusions

Participation in a prehabilitation and recovery programme may confer emotional well-being benefits through a range of mechanisms. Whilst referral to specialist psychological support may be valuable when appropriate for an individual, support implicitly embedded within a largely exercise-focussed programme appeared to yield valued benefits.

### Electronic supplementary material

Below is the link to the electronic supplementary material.

Appendix: Analysis details

## Data Availability

In order to maintain participant confidentiality, in line with ethical approval and participant consent, this study’s datasets are not publicly available: restricted access only is permissible. Contact the corresponding author for information.

## List of Abbreviations

COVID-19: Coronavirus disease
GM: Greater Manchester
GP: General Practitioner
IMD: Index of Multiple Deprivation
NHS: National Health Service
NICE: National Institute for Health and Care Excellence
P4C Programme: The Greater Manchester Cancer Alliance Prehab4Cancer and Recovery Programme
REC: Research Ethics Committee
