# Supplementary material for: Impact of a prehabilitation and recovery programme on emotional well-being in individuals undergoing cancer surgery: a multi-perspective qualitative study
Source: BMC Cancer. 2023 Dec 14;23:1232. doi: 10.1186/s12885-023-11717-1 (PMC10722769; doi:10.1186/s12885-023-11717-1)
Supplement: Supplementary file 1 — Appendix: Analysis details [file 12885_2023_11717_MOESM1_ESM.docx]

**Impact of a prehabilitation and recovery programme on emotional well-being in individuals undergoing cancer surgery: A multi-perspective qualitative study**

**Appendix: Analysis details**

The analysis followed methods also reported in Powell et al. (2023). Interviews were audio-recorded, professionally transcribed and identifying details removed; field notes were also made after interviews. An inductive thematic analysis was conducted aiming to identify and understand patterns within the dataset (Braun & Clarke, 2006; Spencer, Ritchie, Ormston, O'Connor, & Barnard, 2014). A critical realism perspective was taken: reality was seen to exist outside an individuals’ experience, but accessed via individuals’ interpretations of it (Ormston, Spencer, Barnard, & Snape, 2014). We aimed to understand perspectives of both patient and healthcare staff (‘clinician’) participants, and conducted a multi-perspective analysis, integrating data from the two participant groups. The analysis was structured using the Framework approach (Ritchie & Spencer, 1994; Spencer et al., 2014). The first and second authors led the analysis process, with the involvement of all other authors.

Whilst the Theoretical Framework of Acceptability (Sekhon, Cartwright, & Francis, 2017) was used when developing the patient interview schedule, it was not used as a basis for conducting the analysis. The Framework Approach was employed to structure analysis (Ritchie & Spencer, 1994; Spencer et al., 2014). Framework is a systematic and transparent process, which enables other research team members to follow decisions made and processes followed by lead analysts. Early analysis stages were conducted for the two participant groups, with their different perspectives, separately. The two datasets were later brought together within the same analysis, allowing issues to be scrutinised from both healthcare staff and patient perspectives. Analysis was performed using Excel spreadsheets and Word documents.

The first and second authors (RP and AD) read and re-read patient interview transcripts, noting potential issues of importance, to familiarise themselves with the dataset. The first three interviews were coded by both RP and AD: labels were develop to capture ideas identified within interviews. RP and AD discussed these codes and used them to develop an early-stage coding framework for patient data. RP coded all remaining transcripts and further developed the coding framework. RP and AD met weekly to discuss the analysis process and framework development, until a working coding framework – a hierarchical list of categories and sub-categories of issues identified within the dataset - was produced. This document was discussed with all authors to gain their thoughts on the analysis. The coding framework is available elsewhere (Powell et al, 2023).

RP conducted familiarisation and coding of healthcare staff survey responses. After careful reading of all responses, the first nine survey response sets were coded and used in developing an early-stage coding framework. The additional surveys were re-read; additional codes were identified and brought into the working coding framework as/when appropriate.

RP then combined the coding frameworks from patient and healthcare staff datasets, resulting in one coding framework which reflected thoughts and issues identified across the full dataset. A sample of healthcare staff surveys was read by AD; RP and AD then discussed and refined the coding framework. RP then ‘indexed’ the data: applied this coding framework to both patient and clinician datasets.

RP developed ‘charts’: matrices containing framework categories in columns and participants in rows. Cells contained a summary of the essence of participant responses related to the specific framework category. Line numbers were included in entries, so that the researchers could consistently work with transcripts, and not lose the context within which participants expressed ideas. These charts were then examined, with the analysis aiming to understand the experiences and perspectives of participants within categories, and to understand how content in different categories related to each other, within and between participants. During this process, there was some re-ordering of chart columns, such that related content and ideas could be brought more closely together. This re-ordering facilitated the final analysis stage: writing. RP worked back and forth across charts, patient participant interview transcripts and healthcare staff survey responses to develop themes, to review chart summaries and potential quotes in the context of the interviews and surveys, and increase understanding of issues. Preliminary findings were shared with all authors for discussion, before being refined, yielding final analyses.

The online surveys completed by healthcare staff participants included categorical, tick-box questions alongside free-response boxes. Responses to these categorical questions were summarised numerically and are presented elsewhere (Powell et al., 2023).

In developing our analysis strategy, we were mindful of the roles held by various members of the research team. The research-focussed team members (AD, RP and DF) were independent of the GM Cancer Prehab4Cancer and Recovery (P4C) Programme, whilst JM, ZM and KRG had key roles within the P4C Programme. The wider study resulted from JM and ZM wishing to identify and address any areas of the programme which could be improved, rather than their seeking approval of the programme, but we recognised the value of independent researchers leading the research processes to minimise any risk of developing overly positive conclusions. At the same time, because the research-focussed team members were not involved in developing or delivering the P4C Programme, they did not have the understanding of contextual issues around P4C delivery that JM, ZM and KRG had. We sought to minimise risk of bias in interpreting data whilst making use of knowledge of all research team members by having the analysis process led, and conducted, by research-focussed team members RP and AD. Where RP and AD were uncertain of contextual issues impacting understanding of the data, they were able to ask ZM, KRG and JM for information to address their knowledge gaps. The full research team was consulted at key stages: a draft working analytic framework and a preliminary findings document were shared with all members of the research team for discussion, and all research team members reviewed and approved the final submitted paper. This enabled insights from all perspectives to be drawn on to ensure as full an understanding of issues raised as possible, whilst still keeping control of the analysis with the research-focussed team members.

Transcripts and analysis documents were not presented to research participants for member checking. We wished to minimise participant burden, and the evidence around the value of member checking seems unclear (Thomas, 2017).

**References**

Braun, V., & Clarke, V. (2006). Using thematic analysis in psychology. *Qualitative Research in Psychology, 3*, 77-101.

Ormston, R., Spencer, L., Barnard, M., & Snape, D. (2014). The foundations of qualitative research. In J. Ritchie, J. Lewis, C. McNaughton Nicholls, & R. Ormston (Eds.), *Qualitative Research Practice: A Guide for Social Science Students & Researchers* (pp. 1-25). London: SAGE Publications Ltd.

Powell, R., Davies, A., Rowlinson-Groves, K., French, D. P., Moore, J., & Merchant, Z. (In Press). Acceptability of prehabilitation for cancer surgery: A multiperspective qualitative investigation of patient and 'clinician' experiences. *BMC Cancer, 23, 744.* https://doi.org/10.1186/s12885-023-10986-0

Ritchie, J., & Spencer, L. (1994). Qualitative data analysis for applied policy research. In A. Bryman & R. G. Burgess (Eds.), *Analysing Qualitative Data* (pp. 173-194). London: Routledge.

Sekhon, M., Cartwright, M., & Francis, J. (2017). Acceptability of healthcare interventions: An overview of reviews and development of a theoretical framework. *BMC Health services Research, 17*, 88. doi:10.1186/s12913-017-2031-8

Spencer, L., Ritchie, J., Ormston, R., O'Connor, W., & Barnard, M. (2014). Analysis: principles and processes. In J. Ritchie, J. Lewis, C. McNaughton Nicholls, & R. Ormston (Eds.), *Qualitative Research Practice: A Guide for Social Science Students & Researchers* (pp. 267-293). London: SAGE Publications Ltd.

Thomas, D. R. (2017). Feedback from research participants: Are member checks useful in qualitative research? *Qualitative Research in Psychology, 14*, 23-41. doi:10.1080/14780887.2016.1219435
